# Catalytic degradation of diclofenac from aqueous solutions using peroxymonosulfate activated by magnetic MWCNTs-CoFe_3_O_4_ nanoparticles

**DOI:** 10.1039/c9ra02757b

**Published:** 2019-05-28

**Authors:** Yousef Dadban Shahamat, Mohammad Ali Zazouli, Mohammad Reza Zare, Nezamaddin Mengelizadeh

**Affiliations:** Environmental Health Research Center, Department of Environmental Health Engineering, Faculty of Health, Golestan University of Medical Sciences Gorgan Iran; Department of Environmental Health Engineering, Health Sciences Research Center, Faculty of Health, Mazandaran University of Medical Sciences Sari Iran; Department of Environmental Health Engineering, Evaz Faculty of Health, Larestan University of Medical Sciences Larestan Iran; Research Center of Health, Safety and Environment, Department of Environmental Health Engineering, Evaz Faculty of Health, Larestan University of Medical Sciences Larestan Iran nezam_m2008@yahoo.com +98-939-231-2472

## Abstract

CoFe_3_O_4_ nanoparticles supported on multi-walled carbon nanotubes (MWCNTs-CoFe_3_O_4_) were synthesized by the co-precipitation method as a novel catalyst for degradation of diclofenac (DCF). The comparative experiments indicated that MWCNTs-CoFe_3_O_4_ has a better catalytic activity in degradation of DCF and activation of peroxymonosulfate (PMS) compared to other catalytic systems. This can be attributed to the interaction of MWCNTs with CoFe_3_O_4_ in accelerating the absorption process and activating the PMS (*E*_a_ = 22.93 kJ mol^−1^). The removal efficiencies of DCF and total organic carbon (TOC) were 99.04% and 50.11%, under optimum conditions, *e.g.*, pH of 7, PMS dosage of 4 mM, DCF concentration of 30 mg L^−1^, catalyst dosage of 500 mg L^−1^, and reaction time of 120 min. The oxidation of DCF was fitted by the pseudo-first-order kinetic model and the constant rate was increased by increasing the pH, temperature, dosage of PMS and catalyst. The production of reactive species was studied using scavengers such as TBA and ethanol and the results showed that sulfate radical is the reactive species responsible for the degradation of DCF. The MWCNTs-CoFe_3_O_4_ catalyst showed high stability and reusability based on five successful repeated reactions, X-ray diffraction and energy dispersive X-ray spectroscopy analysis. Based on the intermediates detected by gas chromatography-mass spectrometry (GC-MS), the possible pathways for DCF catalytic oxidation were proposed. The results explained that the PMS/MWCNTs-CoFe_3_O_4_ system is a promising method for treating DCF solution due to high efficiency, good reusability of catalyst and greater PMS activation.

## Abstract 2

The MWCNTs-CoFe_3_O_4_ as a novel catalyst showed high catalytic activity in activation of proxymonosulfate for degradation of diclofenac.

## Graphical abstract

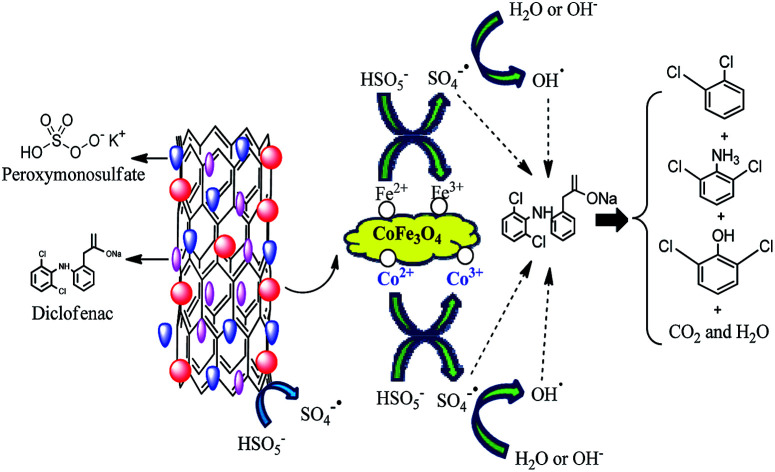

## Introduction

Over the past decades, pharmaceuticals and personal care products have been widely found as emerging contaminants due to their widespread use for the treatment of human and animal diseases.^1^ The worldwide consumption of drugs releases high amounts of the original compound or their metabolites into the environment. Recent studies towards the occurrence of these organic compounds indicate the presence of high amounts of pharmaceuticals belonging to different therapeutic groups in wastewater, surface water and groundwater due to their polar non-volatile nature.^1,2^ The presence of such compounds in aquatic environments for a long time may result in bioaccumulation and negative effects on aquatic ecosystems and human health.^3^ Diclofenac (DCF, 2-(2,6-dichlorophenylamino) phenylacetic acid) is one of the most important non-steroidal anti-inflammatory drugs (NSAIDs) with a total consumption of 1014 tons to treat inflammation and pain associated with rheumatic and non-rheumatic diseases.^4,5^ Due to its high stability and lower removal in sewage treatment plants, DCF has been found at a range from ng L^−1^ to μg L^−1^ in surface waters and groundwater.^4–6^ The accumulation of DCF in the environment or the food chain has toxic effects on various environmental species.^7,8^ Therefore, an effective method is needed to remove this drug from aqueous solutions.

In recent years, advanced oxidation processes based on the production of sulfate radical 
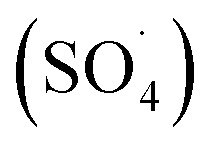
 have considered as a promising method for the removal of persistent organic compounds due to high redox potential (2.5–3.1 V) and application in a wide range of pH.^4,9^ Compared to hydroxyl radical (·OH), 
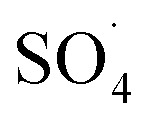
 has high selectivity and long lifetime (·OH: <1 μs; 
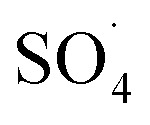
: 30–40 μs).^9,10^ This reactive species can be produced *via* activating the persulfate or PMS by heat, UV, and metal ions such as cobalt, iron, and copper.^10^ Activation by transition metal such as cobalt (Co) has been widely investigated for the removal of various pollutants due to high reactivity, low cost and the lack of need for a complex reactor compared to energy-based activation methods.^9,11^ However, the application of the PMS/Co process has been limited due to the need for high dosage in the treatment of strong wastewater, hard separation and cobalt ion toxicity.^12,13^ To avoid problems associated with the PMS/Co homogeneous system, researchers have recently proposed cobalt loading on the solid support such as zeolite,^14^ activated carbon,^15^ graphene,^16^ alumina^17^ and TiO_2_^18^.

Compared to the support materials mentioned above, carbon nanotubes (CNTs), as seamless cylinders of single or multi-layer graphene, have good physicochemical properties such as high surface area, good chemical stability, high thermal stability and good chelating with metals.^19–22^ These unique features have increased the tendencies for the application of CNT as metal supporting to remove various pollutants.^23^ Su *et al.* coated the Cu_3_N nanocrystal on carbon nanotubes and found that CNTs with a high specific surface area and high electronics activity facilitated the transfer of electrons for the oxygen reduction reaction by Cu_3_N crystals.^24^ Pourzamani *et al.* reported that the presence of CNTs in the composite MWCNTs-Fe_3_O_4_, in addition to preparing the required substrate for adsorbing and oxidizing the pollutants, produces more reactive oxygen species (ROSs) through the electrochemical reduction of oxygen.^21^ However, PMS/CNTs-Co systems require the isolation or precipitation processes, which limit its full-scale application.^25^ In order to overcome these limitations, the magnetization of the composite was suggested by the researchers.^26^ Magnetic separation, due to easy recycling of the catalyst, lack of need for a centrifuge stage and the development of the specific surface area of catalyst for adsorption and oxidation, is considered as the efficient and rapid technology.^27^ In addition, some studies have reported the use of Fe_3_O_4_ for activation of PMS to produce ROSs.^28^ Liu *et al.* investigated the activation of PMS by the MnO_2_–Fe_3_O_4_ nanocomposite and observed that, by converting Fe^2+^ to Fe^3+^, the sulfate radical is produced by PMS activation.^29^ Tan *et al.* also reported that Fe^2+^–Fe^3+^ on the catalyst surface is the main factor for the activation of PMS and the production of ·OH and 
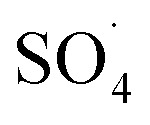
.^30^

In spite of the good performance reported for CNTs and Fe_3_O_4_ and significant risk of DCF in aqueous solutions, removal of the DCF with 
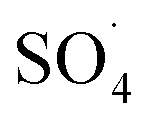
 produced from activation of PMS using the MWCNTs-CoFe_2_O_3_ composite has scarcely studied. Therefore, in this study, the MWCNTs-CoFe_3_O_4_ catalyst was synthesized by the co-precipitation method and its physicochemical properties were determined by various techniques such as field emission scanning electron microscopy, transmission electron microscopy, X-ray diffraction, Fourier transform infrared spectroscopy and energy-dispersive X-ray spectroscopy. The performance of the PMS/MWCNTs-CoFe_3_O_4_ system in DCF degradation was evaluated by studying important parameters such as initial pH, DCF concentration, PMS dosage, catalyst dosage, solution temperature and presence of various anions. Stability and reusability of the catalyst were investigated in five run cycles reaction. The reactive species were investigated by studying the catalytic activity of the process and the scavenging test. Finally, the intermediates of DCF degradation were identified by GC-MS and its degradation pathways were suggested.

## Experimental

### Material and regents

Potassium peroxymonosulfate (oxone, 2KHSO_5_·KHSO_4_·K_2_SO_4_, >98%), iron(iii) chloride hexahydrate (FeCl_3_·6H_2_O, 98%), cobalt(ii) nitrate hexahydrate (Co(NO_3_)_2_·6H_2_O, 98%), sodium hydroxide (NaOH, >97%), chloroform (CHCl_3_, HPLC grade, >99.9%), acetone (CH_3_COCH_3_, HPLC grade, >99.8%), and *N*-trimethylsilyl *N*-methyl trifluoroacetamide (MSTFA, CF_3_CON (CH_3_)Si(CH_3_)_3_, >98%) was purchased from Sigma Aldrich. Diclofenac sodium salt (C_14_H_10_Cl_2_NNaO, 98%) was prepared from Sigma Aldrich as the studied pollutant. Multi-walled carbon nanotubes (MWCNTs, >110 m^2^ g^−1^, 95%) were purchased from US research nanomaterials (Houston, USA). All solutions were prepared by purified water.

### Synthesis of MWCNTs-CoFe_3_O_4_

The MWCNTs-CoFe_3_O_4_ nanoparticles were prepared, according to the co-precipitation method, reported by Oh *et al.* with some modifications.^31^ First, 0.45 g FeCl_3_·6H_2_O and 0.25 g Co (NO_3_)_2_·6H_2_O were dissolved in 100 mL distilled water. 0.5 g of MWCNTs was added to the stirred solution with a magnetic stirrer for 1 h. The pH of the solution was then adjusted to pH of 10–11 using 1 M NaOH. The solution was stirred for 2 h at 100 °C. After cooling the solution, the solid product was isolated using a magnetic field and washed several times with distilled water. Finally, the solid product was dried at 60 °C for 12 h. MWCNTs-Fe_3_O_4_ nanoparticles were prepared under the same conditions without the addition of cobalt.

### Characteristics

The structure and morphology of nanoparticles were determined by field emission scanning electron microscopy (FESEM, MIRA III model, TESCAN, Czech) and high-resolution transmission electron microscope (TEM, JEM-3000F). The crystalline structure of the synthesized catalyst was determined using an X-ray diffractometer using a Cu Kα radiation source which operated at 45 kV and 40 mA. The chemical compositions of the catalyst were analyzed by energy-dispersive X-ray (EDX) analysis coupled with MIRA III SEM. Functional groups and chemical bands were evaluated by Fourier transform infrared spectroscopy (FTIR, AVATAR model, USA) with a frequency range of 400–4000 cm^−1^. The point of zero charge of the nanoparticles was determined using the Zetasizer Nano analyzer (Malvern, UK).

### Catalytic degradation of DCF

Diclofenac degradation experiments were performed in 250 mL conical flask containing 100 mL of the aqueous solution. An appropriate dose of MWCNTs-CoFe_3_O_4_ and PMS was added to the flask containing a specific concentration of DCF solution. At different times, a sample was taken from the flask for analysis using GC-MS. The solution pH was adjusted by sulfuric acid (0.1 M) and sodium hydroxide (0.1 M). The effect of coexisting anions on DCF removal was studied in the presence of NaCl, NaNO_3_ and NaHCO_3_. Quenching experiments were performed under the optimum conditions with *tert*-butyl alcohol (TBA) and methanol agents. The stability and reusability of nanoparticles were studied by repeated experiments under similar optimum conditions.

### Analytical methods

The concentrations of DCF and its intermediates were measured by a GC-MS system (Agilent, USA) equipped with a DB-5 column. COD values were measured by low and high range COD ampoules with a spectrophotometer (DR 5000, HACH). Total organic carbon (TOC) was analyzed by Shimadzu TOC Analyzer. The removal efficiency (*R*) was calculated by eqn (1). Kinetics of DCF removal was described by the pseudo-first-order equation (eqn (2)).1
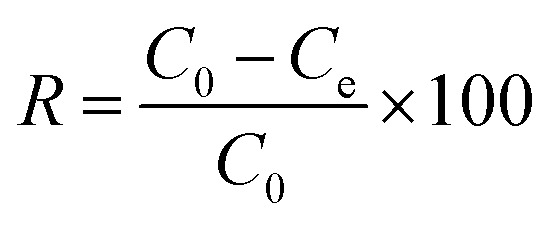
2
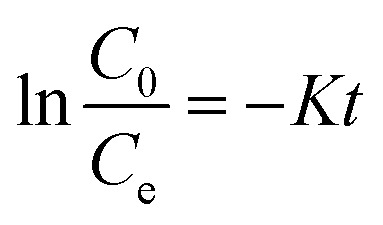
where *C*_0_ and *C*_e_ are the initial and final concentrations of DCF, respectively and *K* is kinetic constant rate.

## Results and discussion

### Characteristics of MWCNTs-CoFe_3_O_4_

**Fig. 1:**
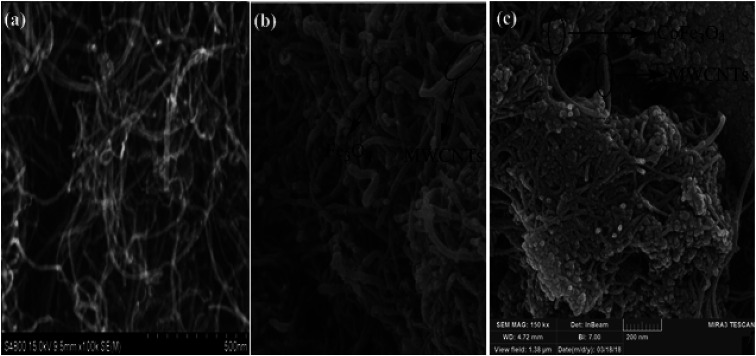
SEM images of MWCNTs (a), MWCNTs-Fe_3_O_4_ (b) and MWCNTs-CoFe_3_O_4_ (c).

**Fig. 2:**
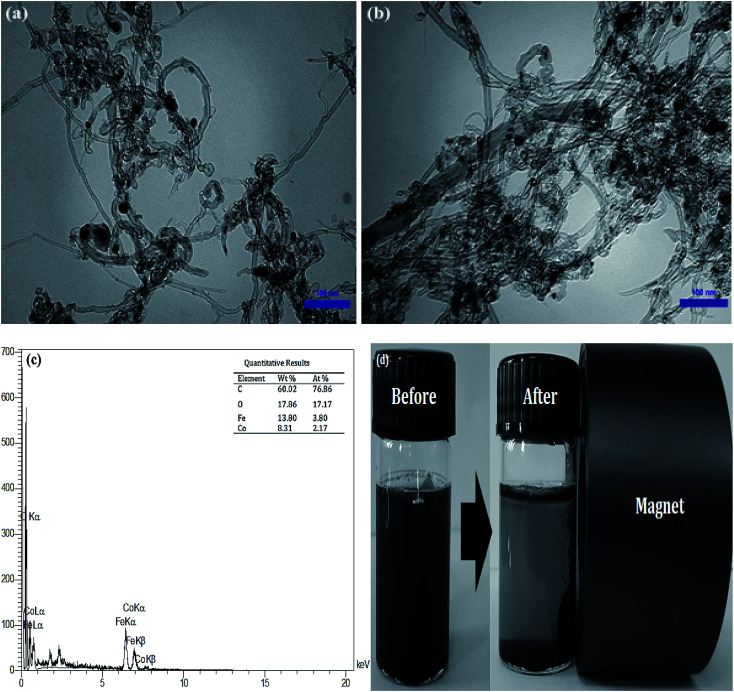
TEM images of MWCNTs-Fe_3_O_4_ (a) and MWCNTs-CoFe_3_O_4_ (b); EDX spectra of MWCNTs-CoFe_3_O_4_ (c); magnetic properties of MWCNTs-CoFe_3_O_4_ (d).

The morphology of MWCNTs and synthesized catalysts, *i.e.*, MWCNTs-Fe_3_O_4_ and MWCNTs-CoFe_3_O_4_ have been obtained by SEM images and the results are shown in Fig. 1. It was observed that the Fe_3_O_4_ and CoFe_3_O_4_ nanoparticles were non-uniformly coated on the outer walls of the MWCNTs. In Fig. 2, TEM images showed the distribution of Fe_3_O_4_ and CoFe_3_O_4_ nanoparticles on the surface of MWCNTs. In addition, the EDX spectrum (Fig. 2c) confirmed the presence of iron, cobalt, carbon and oxygen in the prepared MWCNTs-CoFe_3_O_4_ nanoparticle. Fig. 2d confirms the magnetic properties of MWCNTs containing CoFe_3_O_4_ nanoparticles.

**Fig. 3:**
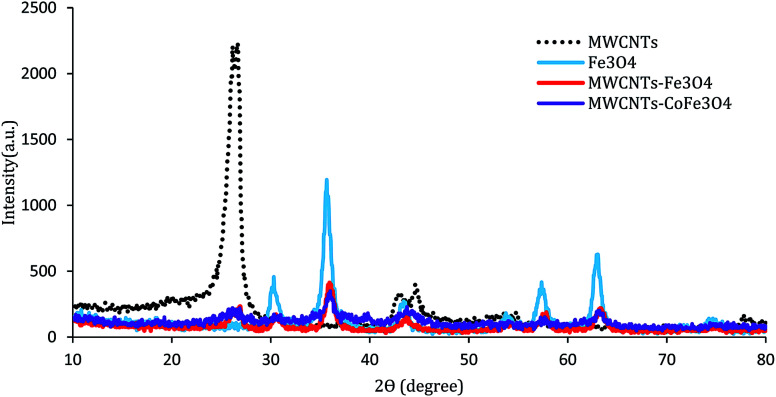
XRD patterns of MWCNTs, Fe_3_O_4_, MWCNTs-Fe_3_O_4_ and MWCNTs-CoFe_3_O_4_.

The XRD pattern of the MWCNTs, MWCNTs-Fe_3_O_4_, and MWCNTs-CoFe_3_O_4_ is shown in Fig. 3. As can be seen, in each of the three samples, there are two characteristic sharp peaks at 2*θ* = 26.13° and 43.98° which are related to the structure of the MWCNTs. For MWCNTs-Fe_3_O_4_, the diffraction peaks observed at 2*θ* = 55.30°, 35.86°, 43.71°, and 57.88° are related to Fe_3_O_4_ nanoparticles.^26,27^ These results were confirmed by the XRD peaks of Fe_3_O_4_ nanoparticles shown in Fig. 3. Compared to the catalysts mentioned above, the diffraction peaks of the MWCNTs-CoFe_3_O_4_ were broad, which indicates iron and cobalt are coated on MWCNTs.

**Fig. 4:**
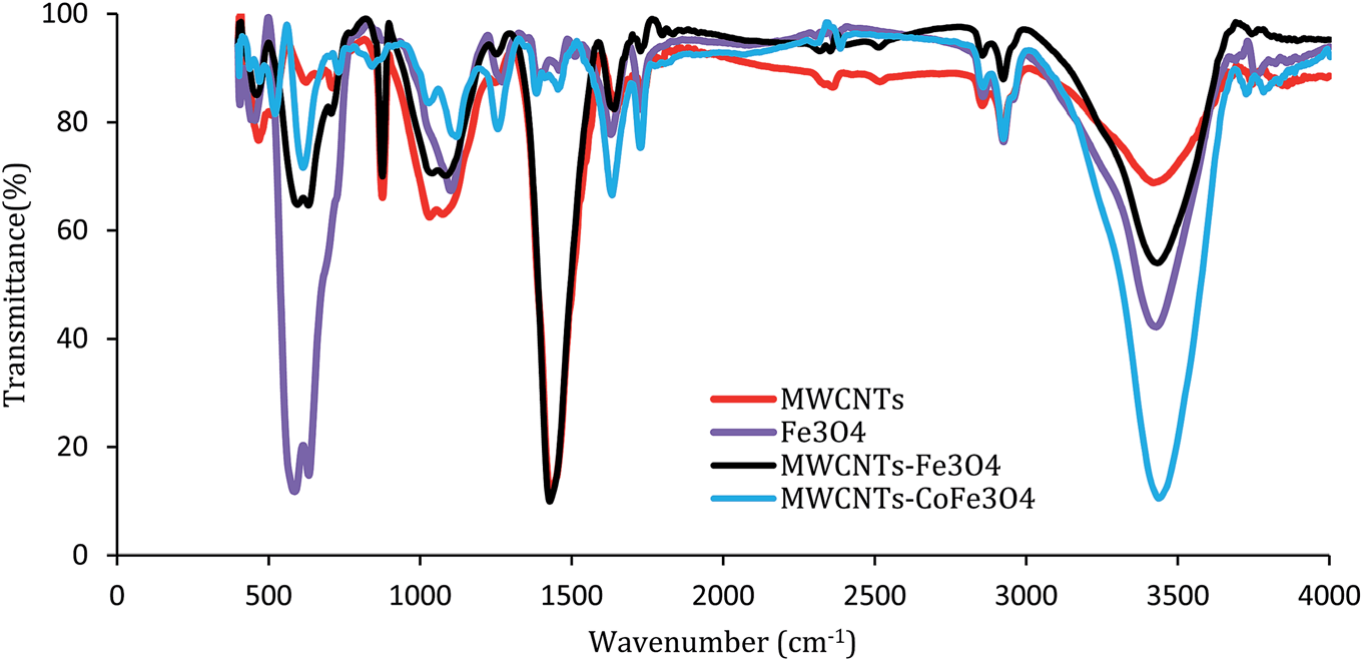
FTIR spectrum of MWCNTs, Fe_3_O_4_, MWCNTs-Fe_3_O_4_ and MWCNTs-CoFe_3_O_4_.

Fig. 4 shows the FTIR absorption spectra of samples recorded in the range of 400–4000 cm^−1^. For MWCNTs, the bands of 1050 cm^−1^, 1430 cm^−1^, 1600 cm^−1^, 2900 cm^−1^, and 3450 cm^−1^ are related to stretching modes and bending of C–O, C–C, C–O, –CH_2_ and –OH. For the FTIR spectrum of the MWCNTs-Fe_3_O_4_ nanoparticle, except for the mentioned bands, there is a strong IR band near the wavelength of 570 cm^−1^, which is related to the Fe–O characteristic peak.^26^ These results were confirmed by the FTIR spectra of Fe_3_O_4_ nanoparticles shown in Fig. 4. In contrast, the MWCNTs-CoFe_3_O_4_ spectra were weaker and smoother at some wavelengths, which is indicative of the changes in the MWCNTs surface due to the co-precipitation process. In this FTIR, strong absorption was observed between 600–500 cm^−1^ compared to MWCNTs, which indicates the formation of CoFe_3_O_4_ on the surface of MWCNTs.^32–34^

### The catalytic activity of MWCNTs-CoFe_3_O_4_

**Fig. 5:**
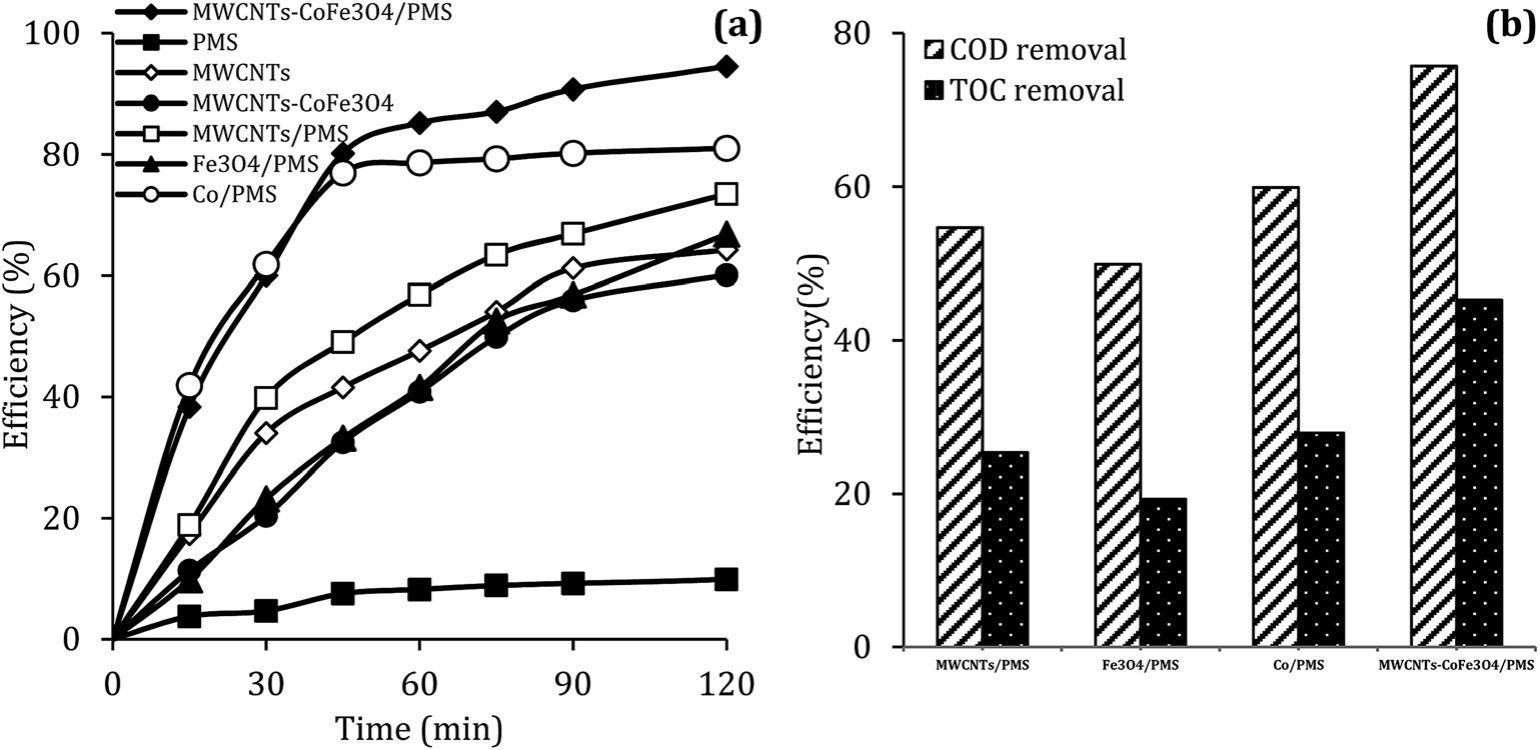
The removal efficiency of DCF (a), COD and TOC (b) under different conditions (reaction conditions: pH = 7, MWCNTs-CoFe_3_O_4_ = 200 mg L^−1^, Co^2+^ = 16.5 mg L^−1^, PMS = 2 mM, DCF = 30 mg L^−1^).

To evaluate the DCF catalytic degradation by MWCNTs-CoFe_3_O_4_, a series of experiments were carried out using different catalysts in two absorption and degradation states. As can be seen in Fig. 5a, a small degradation of DCF occurred in the presence of PMS alone (2 mM). However, the DCF removal efficiency was obtained to be 64.26% and 60.10% using the MWCNTs and MWCNTs-CoFe_3_O_4_, respectively. This efficiency may be due to the high surface area of MWCNTs. A similar trend was reported by Hu and Cheng,^35^ Czech and Oleszczuk^36^ for the adsorption of DCF by MWCNTs. Addition of Fe_3_O_4_ to a solution containing DCF and PMS increased the DCF degradation efficiency to 66.83% at 120 min. Tan *et al.* described this increase in efficiency based on the adsorption capacity of Fe_3_O_4_ nanoparticles and the production of ·OH and 
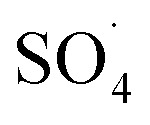
 by the reaction between Fe^2+^ ions on the catalyst surfaces and PMS (eqn (3)–(5)).^30^ Similar results were reported by Zhao *et al.* for the activation of PMS by Fe_3_O_4_ nanoparticles.^37^

It can also be seen in Fig. 5a that the performance of the MWCNTs/PMS system in removal of DCF is higher than the MWCNTs alone. This can be due to the production of reactive species through the reaction between PMS and CNTs. Similar results were reported by Chen *et al.*^38^ and Lee *et al.*^39^ about the activation of PMS and persulfate by CNTs, respectively. In addition, the results of Fig. 5a showed that the DCF removal efficiency in the homogeneous PMS/Co system at the initial time (15 and 30 min) was much higher than the MWCNTs-CoFe_3_O_4_/PMS system; but in the higher times, the removal rate was fixed and the complete degradation was not obtained compared to heterogeneous catalyst. In PMS/Co system, homogeneous cobalt can quickly react with PMS and lead to faster production of reactive species. This fast production of radicals results in their loss by reacting with non-target molecules. In contrast, MWCNTs-CoFe_3_O_4_, with controlling the production of radicals during a long period of time, leads to a greater DCF removal than the PMS/Co system. These results emphasize the role of the interaction between MWCNTs, Fe_3_O_4_ and Co on nanocatalysts.

3 Fe(ii) + HSO_5_^−^ → Fe(iii) + SO_5_^2−^ + ·OH

4 Fe(ii) − ^−^OH + HSO_5_^−^ → Fe(ii) − (HO)OSO_3_^−^ + OH^−^

5 Fe(ii) − (HO)OSO_3_^−^ → Fe(ii) − ^−^OH + SO_5_^−^˙

Along with the removal of DCF, the removal rate of COD and TOC in degradation systems at a reaction time of 120 min was investigated. It is clear from the results of Fig. 5b that the removal of COD and TOC using the MWCNTs-CoFe_3_O_4_ system was much higher than other catalytic systems. This high catalytic performance was due to the synergistic effect between Fe_3_O_4_, Co and MWCNTs for activation of PMS and adsorption of pollutant.

### The effect of initial pH

**Fig. 6:**
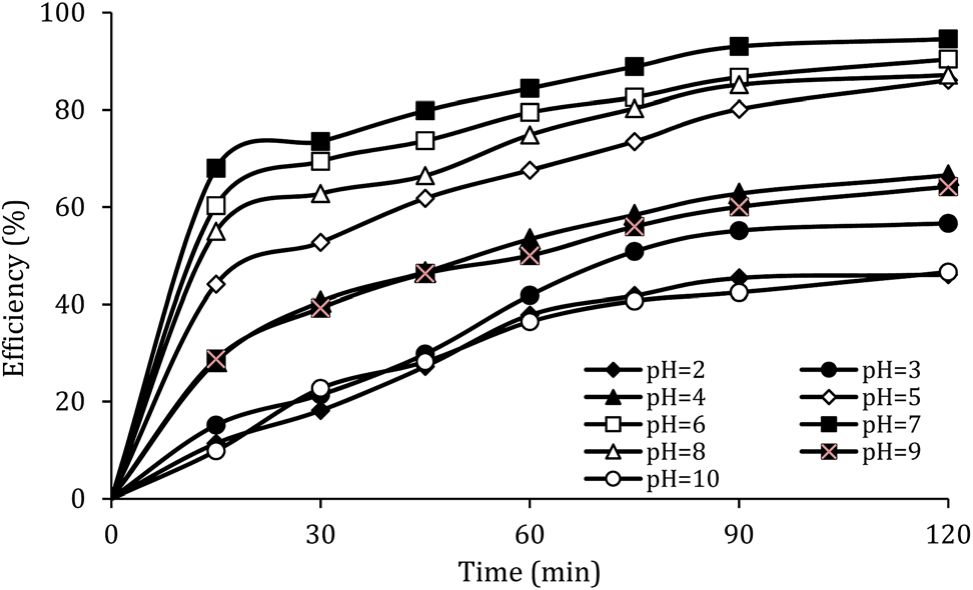
Effect of solution pH on the removal efficiency of DCF.

The solution pH, due to the effect on the properties of the catalyst surface and control of the production rate of reactive species (·OH and 
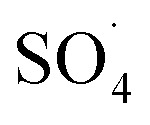
) and the concentration of transition metals (Co and Fe) is one of the important parameters in the PMS activation process.^40^ Therefore, the effect of the solution pH in the range of 2–10 was studied under the condition including MWCNTs-CoFe_3_O_4_ of 200 mg L^−1^, PMS of 2 mM and DCF of 30 mg L^−1^. The DCF removal efficiency gradually increased with an increase in the initial pH and then decreased with a further increase in pH (Fig. 6). The results of the kinetic analysis showed the same trends in DCF removal efficiency (Table 1). The kinetic constant rate was first improved from 0.005 min^−1^ to 0.022 min^−1^, when the initial pH increased from 2 to 7 and it was then decreased to 0.005 min^−1^ with increasing the pH to 10. Increasing the efficiency at pH 7 can be due to the production of the high concentration of 
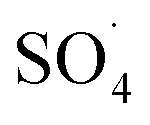
 and ·OH through the reaction of PMS and cobalt ion.^41^ According to Hu *et al.* p*K*_a2_ of H_2_SO_5_ is 9.4, which means that, at the pH values lower than 9.4, PMS is mainly in the form of HSO_5_^−^. Under these conditions, most of the HSO_5_^−^ ions react with cobalt ions (Co^2+^) on the surface of the catalyst and produce high concentrations of reactive species.^42^ In addition, increasing the removal efficiency can be related to the electrostatic adsorption between the positive charge of the MWCNTs-CoFe_3_O_4_ catalyst and the negative charge of DCF. According to the studies, the surface charge of the materials depends on their pH_pzc_ and the solution pH. The surface of the materials is positive when pH_pzc_ is higher than the solution pH and the negative charge is formed when pH_pzc_ is lower than the solution pH.^43^ The zero charge point for catalyst was 8.5. Tabit *et al.* investigated the activation of PMS by CoFe_2_O_4_ nanoparticles supported on graphene oxide, and found that the high degradation efficiency at pH of 7 was due to electrostatic adsorption between the negative charge of the catalyst and the positive charge of rhodamine B.^44^ In addition, Zhang *et al.* observed the same results for removing ibuprofen by activating the PMS using Fe_3_C immobilized on carbon.^45^

**Table 1:** The kinetic parameters for different operating conditions

Parameters	*k*/min^-1^	*R* ^*2*^
pH	Other conditions: catalyst = 200 mg L^−1^, PMS = 2 mM, DCF = 30 mg L^−1^	
2	0.005	0.923
3	0.007	0.949
4	0.008	0.929
5	0.015	0.969
6	0.017	0.904
7	0.022	0.920
8	0.015	0.914
9	0.009	0.919
10	0.005	0.938
Catalyst dosage (mg L^−1^)	Other conditions: pH = 7, PMS = 2 mM, DCF = 30 mg L^−1^	
25	0.010	0.960
100	0.015	0.977
250	0.019	0.969
500	0.024	0.922
750	0.024	0.919
1000	0.015	0.966
PMS dosage (mg L^−1^)	Other conditions: pH = 7, catalyst = 500 mg L^−1^, DCF = 30 mg L^−1^	
0.25	0.009	0.989
0.5	0.011	0.983
1	0.015	0.960
2	0.021	0.922
4	0.032	0.926
6	0.018	0.891
8	0.017	0.986
DCF concentration (mg L^−1^)	Other conditions: pH = 7, catalyst = 500 mg L^−1^, PMS = 4 mM	
10	0.038	0.968
20	0.032	0.923
30	0.032	0.926
50	0.012	0.983
Temperature (°C)	Other conditions: pH = 7, catalyst = 500 mg L^−1^, PMS = 4 mM, DCF = 30 mg L^−1^	
10	0.016	0.985
20	0.018	0.954
25	0.022	0.932
30	0.032	0.926

At lower pH values (<7), decrease in the removal efficiency may be related to the high stability of PMS (eqn (6)) and lack of its proper reaction with metal ions.^16^ In addition, at the acidic pH, the formation of the H bond between the hydrogen ion and the O–O group of PMS is led to decrease the positive charge of PMS, thus hindering the reaction between the PMS and the catalyst surface.^29^ Huang *et al.* investigated the degradation of reactive black B by the PMS/Co process and reported that, by increasing pH from 3.5 to 6, the dye degradation efficiency was increased. They explained the low efficiency in acid pH based on the scavenging effect of the H^+^ ion for 
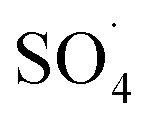
 and ·OH (eqn (7) and (8)).^40^ In addition, similar results were observed in other studies that reported low degradation efficiency under acidic conditions using the graphite felt-Fe_3_O_4_/PMS^46^ and PMS/Co_3_O_4_-graphene processes.^47^

6 Co^2+^ + HSO_5_^−^ ↔ CoSO_5_ + H^+^

7 ·OH + H^+^ + e^−^ → H_2_O

8 SO_4_^−^˙ + H^+^ + e^−^ → HSO_4_^−^˙

At higher pH values (>7), the reduction in efficiency can be due to the electrostatic repulsion between the MWCNTs-CoFe_3_O_4_ catalyst surface and the dominant PMS species as SO_5_^2−^.^48^ In addition, previous studies reported that at alkaline pH, the PMS is decomposed through its non-radical pathways and generates less reactive species.^49^ Xu *et al.* investigated the performance of graphene-CoFe_2_O_4_ as a PMS activator for the degradation of dimethyl phthalate and found that, by increasing the initial pH from 4 to 11, the degradation efficiency of the pollutant is reduced due to the conversion of 
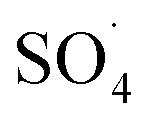
 into oxidants with low oxidation potential such as ·O– (eqn (9) and (10)).^16^ In addition, the findings are similar to those observed in CoFe_2_O_4_/PMS,^50^ ordered mesoporous-MnFe_2_O_4_/PMS^51^ and Al_2_O_3_–CoFe_2_O_4_/PMS processes.^52^

9 SO_4_^−^˙ + OH^−^ → SO_4_^2−^ + ·OH

10 ·OH + H_2_O → ·O^−^ + H_3_O^+^

### Effect of MWCNTs-CoFe_3_O_4_ dosage

**Fig. 7:**
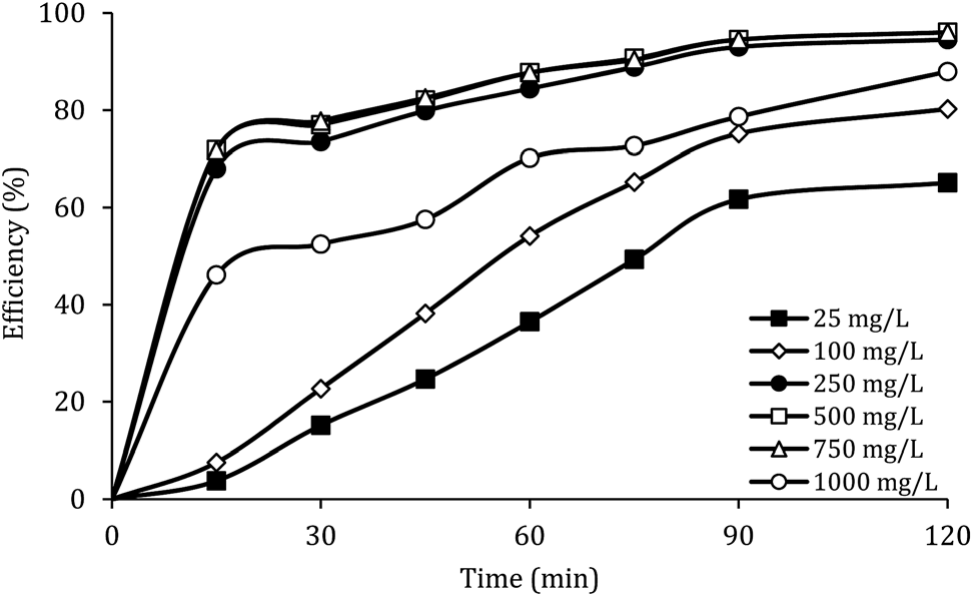
Effect of MWCNTs-CoFe_3_O_4_ dosage on the removal efficiency of DCF.

Fig. 7 shows the effect of the MWCNTs-CoFe_3_O_4_ dosage on the DCF removal efficiency at pH of 7, PMS of 2 mM and DCF of 30 mg L^−1^. According to this figure, by increasing the catalyst dosage from 25 mg L^−1^ to 500 mg L^−1^, the DCF removal efficiency was increased from 65.09% to 96.02% at 120 min of reaction. However, increasing the amount of MWCNTs-CoFe_3_O_4_ to values greater than 1000 mg L^−1^ is led to diminishing the efficiency to 87.93%. Table 1 shows the first-order kinetic constant rate for the effect of the catalyst dosage. As can be seen, at the catalyst dosage of 500 mg L^−1^, the kinetic constant rate was 0.024 min^−1^, while the kinetic rate was obtained to be 0.010 min^−1^ at the catalyst dosage of 1000 mg L^−1^. The increase in removal efficiency can be related to increasing the adsorption sites as well as providing the more active catalytic surface for activation of PMS to produce more active radical species.^29,30^ Decreasing the efficiency can be due to the accumulation of nanoparticles and the scavenging effect of the Fe_3_O_4_ nanoparticles on 
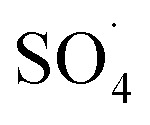
.^29^ Yan *et al.* studied the degradation of trichloroethylene using persulfate activated by graphene oxide/Fe_3_O_4_, and found that, by increasing catalyst dose, degradation efficiency was initially increased and then reduced at the higher catalyst values. The authors explained the reduction in efficiency based on the consumption of 
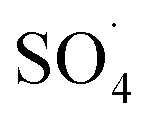
 by extra iron ions (eqn (11)) and persulfate produced in high concentration of sulfate radical (eqn (12) and (13)).^53^ Feng *et al.* reported that by increasing the CuCo_2_O_4_ catalyst dosage to values more than 0.08 g L^−1^, the sulfamethazine degradation efficiency was decreased due to the scavenger effects of Co and Cu on 
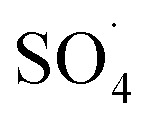
 and ·OH (53).^43^ In addition, similar results were obtained in the degradation of carbamazepine by the Mn_*x*_Co_3−*x*_O_4_ nanocages/PMS system^54^ and the degradation of 4-chlorophenol by the Fe_3_O_4_–MnO_2_/PMS process.^29^

11 Fe^2+^ + SO_4_˙^−^ → Fe^3+^ + SO_4_^2−^

12 SO_4_˙^−^ + SO_4_˙^−^ → S_2_O_8_^2−^

13 SO_4_˙^−^ + S_2_O_8_^2−^ → SO_4_^2−^ + S_2_O_8_˙^−^

### The effect of PMS dosage

**Fig. 8:**
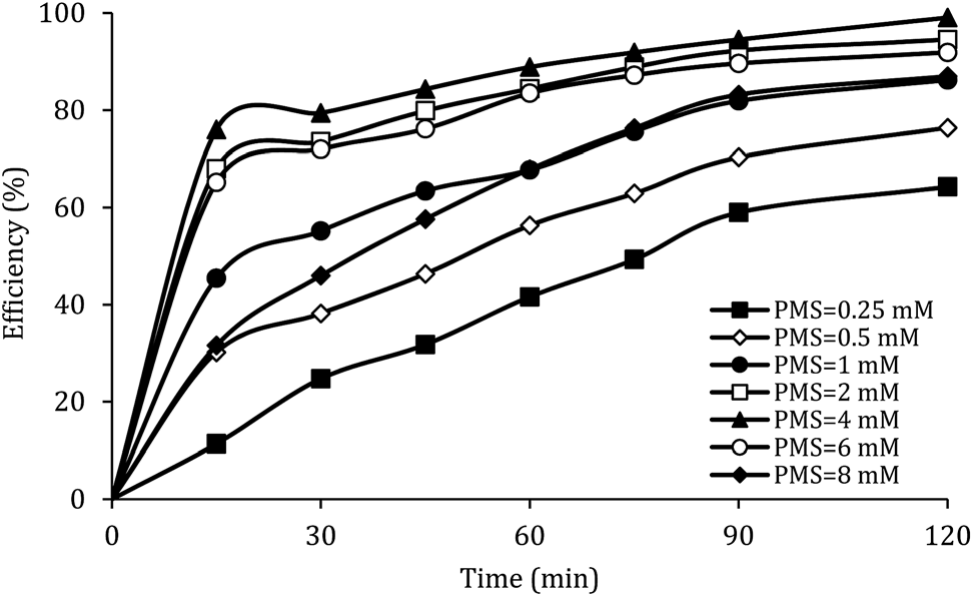
Effect of PMS dosage on the removal efficiency of DCF.

Fig. 8 shows the effect of PMS dosage on DCF removal at pH of 7, MWCNTs-CoFe_3_O_4_ of 500 mg L^−1^, DCF of 30 mg L^−1^. When the PMS dosage increased from 0.25 to 4 mM, the removal efficiency was rapidly increased from 64.26% to 99.047%, and then it was dramatically diminished from 91.86% to 86.95% when the PMS dosage increased from 6 to 8 mM. In Table 1, the kinetic constant rate increased from 0.009 to 0.032 min^−1^ by an increase in PMS dosage from 0.25 to 4 mM, and then decreased with a further increase in PMS to 8 mM. An initial increase in removal efficiency can be due to the production of high levels of 
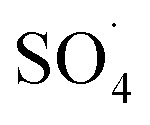
 and ·OH through the reaction of PMS and MWCNTs-CoFe_3_O_4_. However, decreasing the efficiency can be related to the effect of PMS scavenging on 
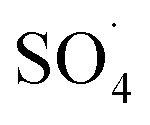
 and ·OH (eqn (14) and (15)).^51,55^ Similar results were observed by Wei *et al.*^56^ and Xu *et al.*^57^ for degradation of dyes using the OMS-Fe_3_O_4_/PMS system and Fe_3_O_4_@C–Co/PMS system, respectively.

14 SO_4_˙^−^ + HSO_5_^−^ → SO_5_˙^−^ + HSO_4_^−^

15 ·OH + HSO_5_^−^ → SO_5_˙^−^ + H_2_O

### Effect of initial concentration of DCF

**Fig. 9:**
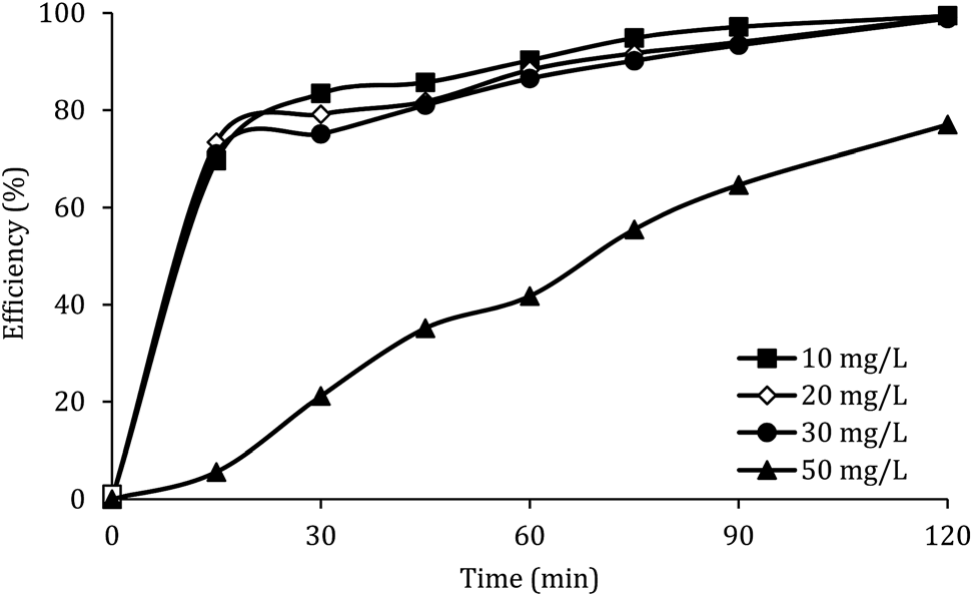
Effect of initial DCF concentration on the removal efficiency of DCF.

The effect of the initial concentration of DCF on the DCF removal efficiency was investigated at pH of 7, MWCNTs-CoFe_3_O_4_ of 500 mg L^−1^ and PMS of 4 mM. As shown in Fig. 9, by increasing the initial concentration of DCF from 10 to 50 mg L^−1^, the DCF removal efficiency was decreased from 99.40% to 76.99% at 120 min. The kinetics analysis results in Table 1 show the same trend in DCF removal efficiency. The kinetic constant rate decreased from 0.038 to 0.012 min^−1^ when the DCF concentration increased from 10 to 50 mg L^−1^. This reduction in efficiency can be related to the low concentration of 
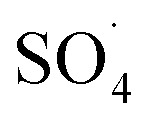
 and ·OH produced as well as the competition between intermediates with main molecules for ROSs.^54^ Tan *et al.* by studying the acetaminophen degradation using the PMS-activated by Fe_3_O_4_ nanoparticles, reported that increasing the concentration of acetaminophen from 2 to 10 mg L^−1^ reduced its degradation efficiency from 100% to 74.7%. The authors explained the decrease in efficiency by this fact that the constant amount of ROSs is produced, when all the factors (pH, PMS, and catalyst) are constant. This number of reactive species produced has the ability to eliminate and to degrade a specific amount of contaminants. Therefore, if the initial concentration of the pollutant is increased, the number of reactive species existed in the environment is not sufficient to remove excessive molecules of contaminant.^30^ In addition, similar results were reported for the degradation of phenol using heterogeneous activation of PMS by graphene-Co_3_O_4_.^58^

Fig. 9 also shows that, at different reaction times, the system efficiency for concentrations of 10 to 30 mg L^−1^ is approximately similar. This finding shows that the process of PMS activated by MWCNTs-CoFe_3_O_4_ is suitable for removal of high concentrations of DCF. A similar trend was reported by Zhou *et al.* for the degradation of 2,4-dichlorophenol.^59^

### The effect of temperature

**Fig. 10:**
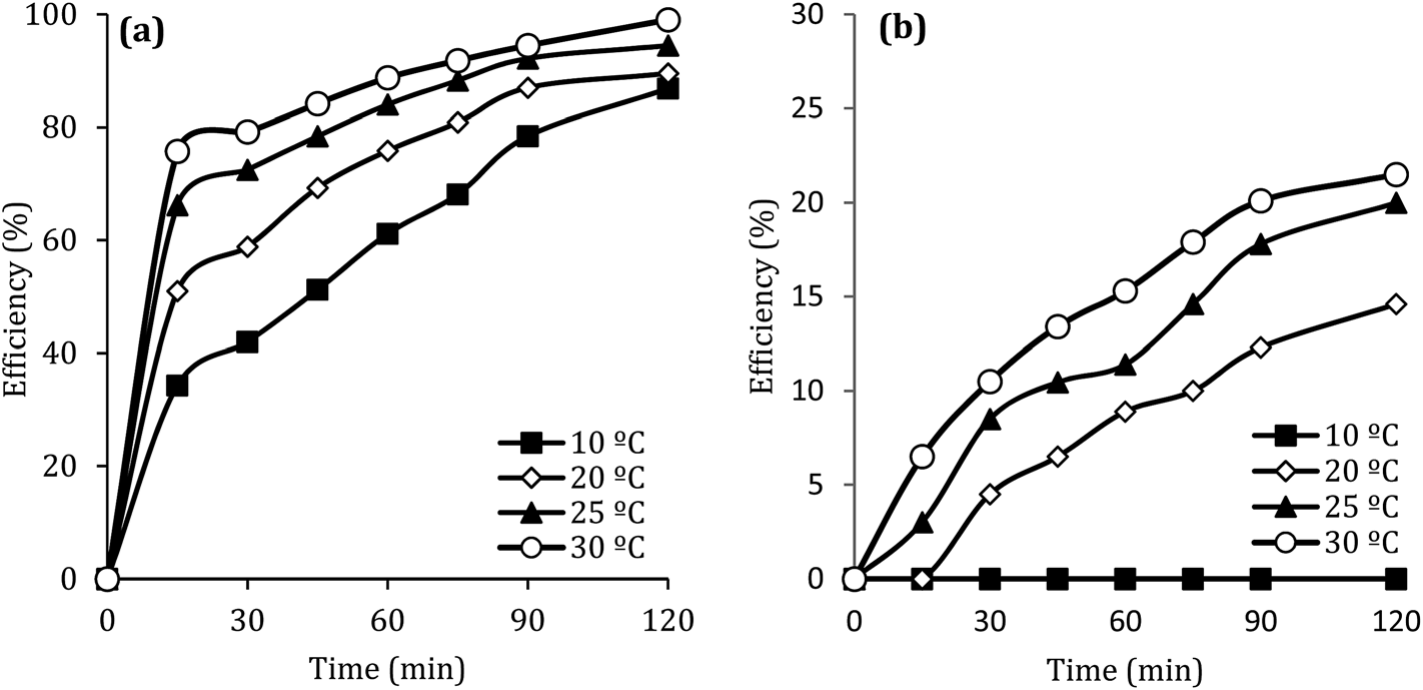
Effect of temperature at presence (a) and without presence of catalyst (b) on the removal efficiency of DCF.

Fig. 10 shows the effect of the solution temperature on DCF removal at pH of 7, MWCNTs-CoFe_3_O_4_ of 500 mg L^−1^, PMS of 4 mM, and DCF of 30 mg L^−1^. As shown in Fig. 10a, with increasing the temperature from 10 to 30 °C, the efficiency of DCF removal increases from 86.83% to 99.04% at the reaction time of 120 min. The kinetic analysis results in Table 1 confirmed this trend. This increase in degradation efficiency can be attributed to decomposing the O–O band in PMS and producing more 
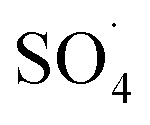
 and ·OH. To further confirm the results, the effect of temperature without catalyst at various temperatures was investigated. The results showed that by increasing the temperature from 10 to 30 °C, the DCF removal efficiency was increased from 0 to 21.5% at the reaction time of 120 min (Fig. 10b). Similar results were obtained in the study of carbamazepine degradation using PMS activated by Mn_*x*_Co_3−*x*_O_4_ nanocages.^54^

The relationship between constant rate and temperature can be evaluated by plotting ln (*K*) *versus* 1/*T*, according to the Arrhenius equation (ln *K* = ln *A* − *E*/*RT*). In this equation, *K* is the constant rate, *R* is the global gas constant (8.314 kJ mol^−1^), *T* is the temperature, *E*_a_ is the activation energy (kJ mol^−1^) and *A* is constant. Linking the *k* values of the first order kinetic and the Arrhenius equation, PMS activation energy by MWCNTs-CoFe_3_O_4_ was found to be 22.93 kJ mol^−1^. This amount of *E*_a_ calculated in this study was much lower than the *E*_a_ values reported in other studies (Table 2), which represent a promising catalytic material for the oxidation processes of pharmaceutical compounds, especially DCF. Table 2 also shows that the low dosages of MWCNTs-CoFe_3_O_4_ at neutral pH have a catalytic activity similar to other catalysts used in the activation of PMS. Therefore, the PMS/MWCNTs-CoFe_3_O_4_ system can be used as a new catalytic process for degradation of DCF.

**Table 2:** Activation energies of catalysts with PMS for pollutant removal^a^

Catalysts	Pollutant	Condition	Efficiency (%)	*E*_a_ (kJ mol^−1^)	References
Fe_3_O_4_	Orange G dye	DC = 0.5 g L^−1^, PMS = 1 mM, *C*_p_ = 0.1 mM, pH = 3 and *T* = 30 min	≃100	54.8	60
Co_3_O_4_/carbon-aerogel	Phenol	DC = 0.2 g L^−1^, PMS = 2 g L^−1^, *C*_p_ = 50 ppm, pH = 4 and *T* = 120 min	≃100	62.9	61
Co_3_O_4_/SBA-15	Phenol	DC = 0.2 g L^−1^, PMS = 2 g L^−1^, *C*_p_ = 30 ppm, pH = 4 and *T* = 180 min	≃100	67.4	62
Mn_3_O_4_–rGO	Orange II dye	DC = 0.05 g L^−1^, PMS = 1.5g L^−1^, *C*_p_ = 60 ppm, pH = 7 and *T* = 120 min	≃80	49.5	63
Mn_2_O_3_ cube	Phenol	DC = 0.4 g L^−1^, PMS = 2 g L^−1^, *C*_p_ = 25 ppm, and *T* = 90 min	≃100	61.2	64
CoFe_2_O_4_/TNTs	Phenol	DC = 0.01 g L^−1^, PMS = 3 g L^−1^, *C*_p_ = 20 ppm, and *T* = 60 min	97.2	70.56	65
MWCNTs-CoFe_3_O_4_	DCF	DC = 0.5 g L^−1^, PMS = 4 mM, *C*_p_ = 30 ppm, pH = 7 and *T* = 120 min	99.04	22.93	This work

a *T* = time; DC = dosage of catalyst; *C*_p_ = initial pollutant concentration.

### The effect of anions

**Fig. 11:**
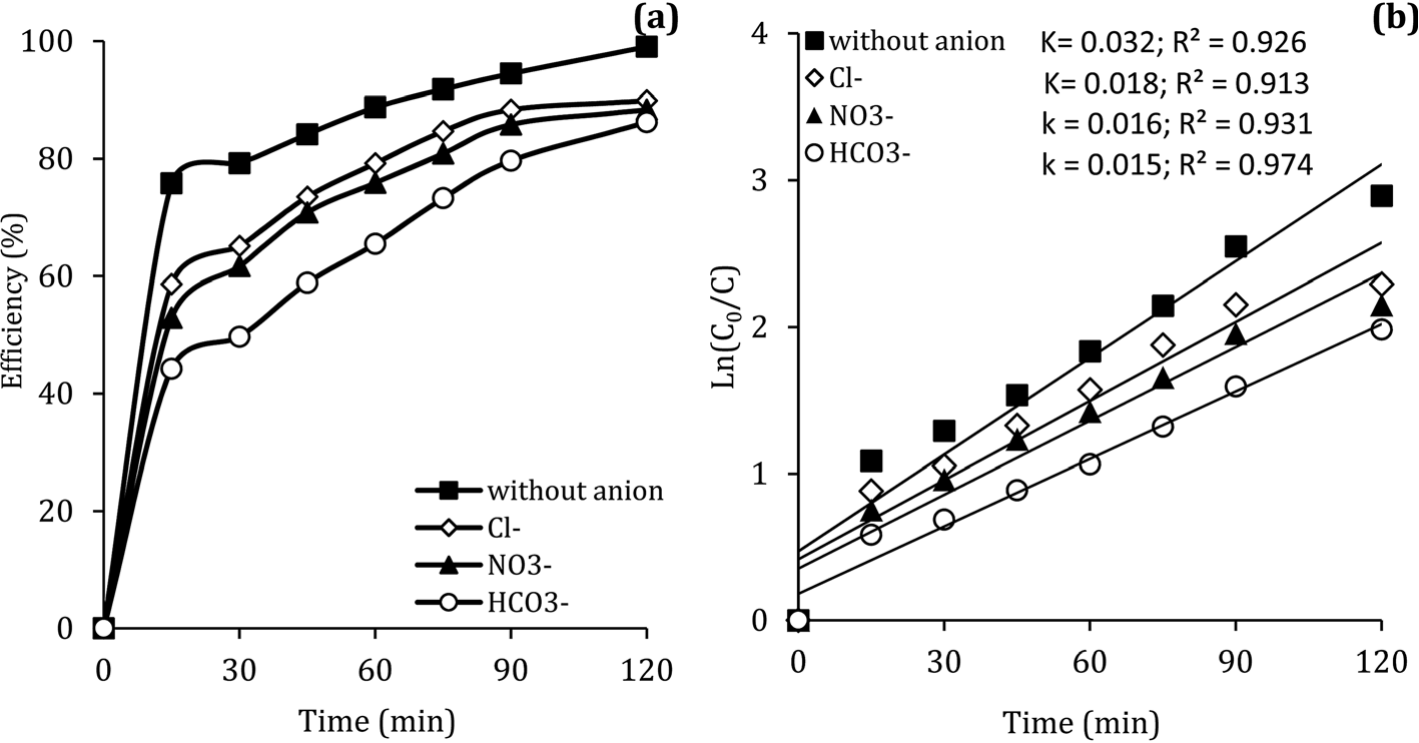
Effect of various anions on the removal efficiency of DCF (a) and kinetic constant rate (b).

In the body of real water, there are various anions, such as bicarbonate (HCO_3_^−^), nitrate (NO_3_^−^) and chloride (Cl^−^), which they can cause the consumption of ROSs and free radical-induced wastewater treatment reaction.^66^ Therefore, in the present study, the DCF removal efficiency in the presence of various anions was investigated at pH of 7, MWCNTs-CoFe_3_O_4_ of 500 mg L^−1^, PMS of 4 mM and DCF of 30 mg L^−1^. As shown in Fig. 11a, the DCF removal efficiency was 89.88%, 88.36% and 86.24% in presence of Cl^−^, NO_3_^−^ and HCO_3_^−^ at 120 min, respectively. The results of Fig. 11b also shows that the kinetic constant rate was decreased from 0.032 min^−1^, at the condition without the presence of anion, to 0.018, 0.016 and 0.015, at the presence of Cl^−^, NO_3_^−^ and HCO_3_^−^, respectively. This could be due to the reaction of anions with 
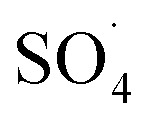
 and the production of reactive species with lower oxidation potential. The similar limiting effect of the anions on the activation of PMS by various catalysts was reported by Zhao *et al.*^67^ and Tan *et al.*^68^

### Reactive species

**Fig. 12:**
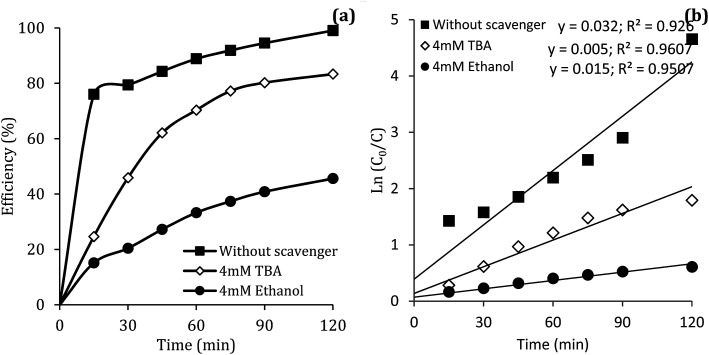
Influence of TBA and ethanol on the removal efficiency of DCF (a) and kinetic constant rate (b).

Previous studies have shown that 
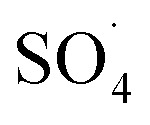
 and ·OH can be produced by homogeneous or heterogeneous activation of PMS. It has also been reported in these studies that TBA is used as ·OH scavenger and ethanol is the scavenger for both 
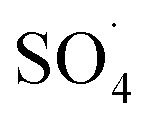
 and ·OH. Thus, in the present study, the reactive species are probed using TBA and ethanol. As can be seen in Fig. 12a, DCF removal is significantly reduced in the presence of scavengers compared to the original system without ethanol and TBA. Moreover, the addition of ethanol has a high limiting efficiency compared to TBA. The results of the kinetic constant rate in Fig. 11b show that the kinetic constant of the ethanol-containing system (0.005 min^−1^) is much lower than the system containing TBA (0.015 min^−1^) and the system without scavengers (0.032 min^−1^). Therefore, the above results suggest that both 
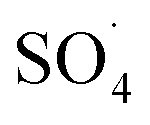
 and ·OH radicals have been produced by PMS activation during the DCF oxidation process and the 
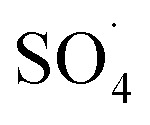
 radical was the main species.

### Reusability of MWCNTs-CoFe_3_O_4_

**Fig. 13:**
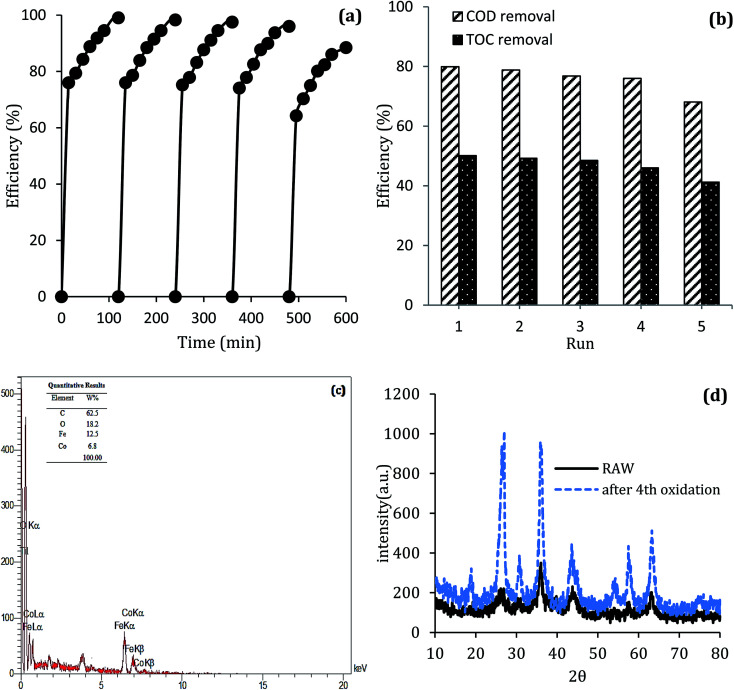
(a) Catalytic performance of recycled MWCNTs-CoFe_3_O_4_ for DCF removal, (b) COD and TOC removal; (c) EDS analysis and (d) XRD pattern of MWCNTs-CoFe_3_O_4_ after the 4th cycle of DCF removal.

The stability of catalyst in catalytic oxidation systems is one of the critical parameters for the ability to recycling the catalyst based on process cost in practical applications. For further studying the stability and reusability of the MWCNTs-CoFe_3_O_4_ catalyst, recycling experiments were performed with centrifuge steps, drying with nitrogen gas and re-dispersing in a DCF-containing solution. As can be seen in Fig. 13a, by increasing the number of runs, the DCF removal efficiency was slowly decreased. After the fourth run, the DCF removal efficiency decreased from 99.04% to 88.46%, while the TOC removal rate decreased from 50.11% to 41.23%. Both of these changes show the reusability of the MWCNTs-CoFe_3_O_4_ catalyst in the heterogeneous activation of PMS. In addition, the decrease in efficiency after the fourth run may be due to leaching of iron and cobalt from the catalyst surface, as well as the occupation of reactive sites of the catalyst by intermediates produced during DCF degradation. The results of Fig. 13c confirmed these results. According to this figure, the weight percentage of iron and cobalt has decreased compared to the raw material synthesized (Fig. 2c).

To investigate the stability of the MWCNTs-CoFe_3_O_4_ catalyst structure, XRD analysis also was performed on the raw material and recycled after four runs of experiments. The results in Fig. 13d show the similar structure and phase of the catalyst before and after the catalytic process. These findings indicate that MWCNTs-CoFe_3_O_4_ has a high potential for reuse and application in catalytic degradation of organic pollutants such as DCF.

### DCF degradation pathways

**Fig. 14:**
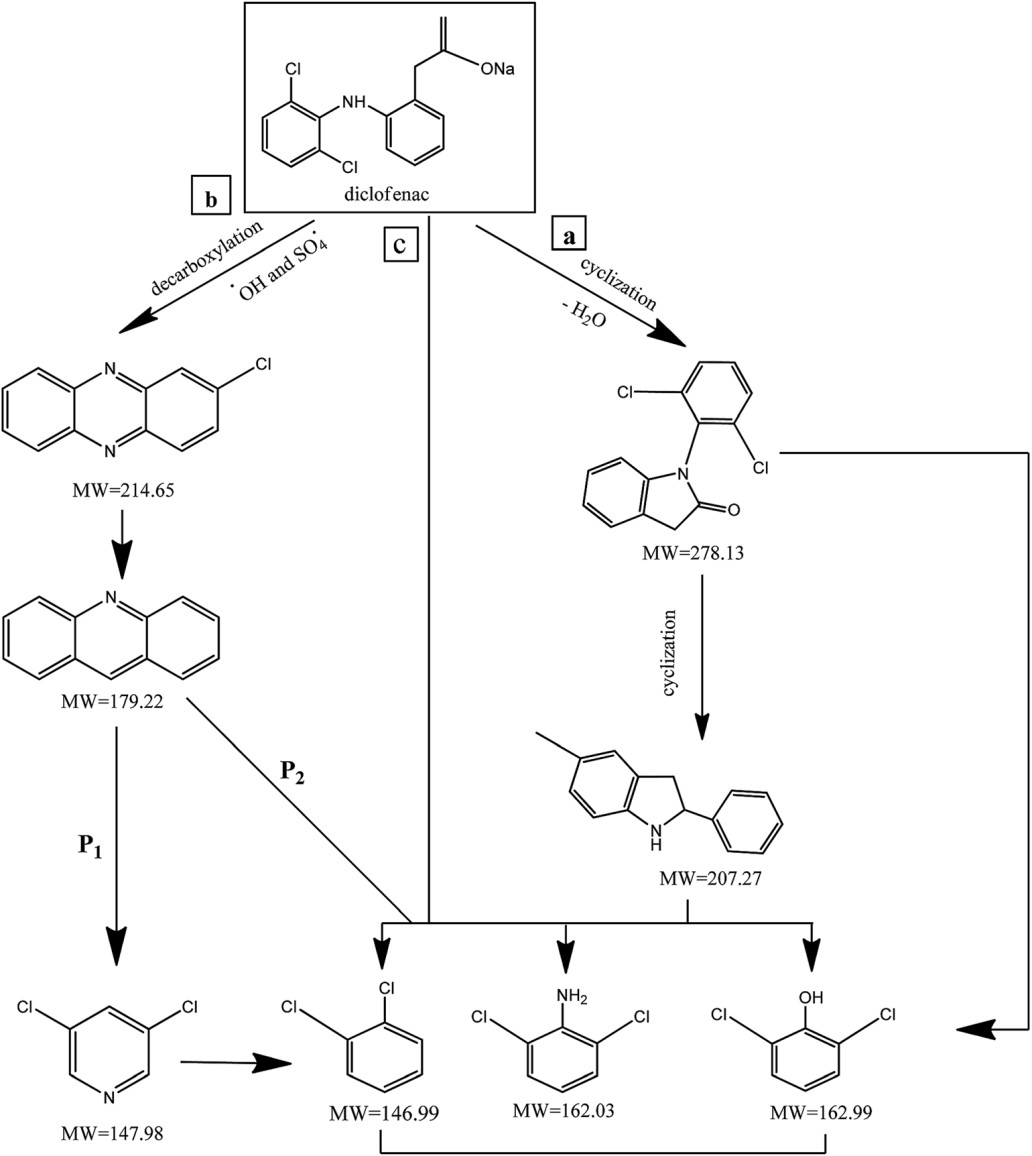
The degradation pathways of DCF in MWCNTs-CoFe_3_O_4_/PMS process.

In order to understand the DCF degradation mechanism through PMS activated by the MWCNTs-CoFe_3_O_4_ catalyst, the intermediates were analyzed using the GC-MS technique. Fig. 14 shows the pathways and byproducts for DCF degradation. According to this figure, there are three reaction pathways for DCF degradation. In pathway a, DCF can be converted to (2,6-diclorophenyl) indolin-2-one through the intramolecular condensation and loss of H_2_O. Then, this byproduct can be transformed into 5-methyl-2-phenyl-1*H*-indole through the cyclization process. Finally, by attack of reactive species (
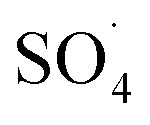
 and ·OH) and cleavage of the C–N bond, 5-methyl-2-phenyl-1*H*-indole is oxidized to 2,6-dichlorophenol, 2,6-dichloroaniline and *O*-dichlorobenzene.

Alternatively, (2,6-dichlorophenyl)-indolin-2-one, during reactive species attack to the C–N chain, may directly lead to 2,6-dichlorophenol, 2,6-dichloroaniline and *O*-dichlorobenzene in the reaction solution. The similar reaction mechanism was reported for DCF degradation by Bae *et al.*^69^ and Pourzamani *et al.*^21^ In the reaction pathway b, 2-chlorophenazine is formed by reactive species attack and decarboxylation process of DCF. This intermediate is then oxidized to 3,4-dichloropyridine (P_1_), and byproducts of 2,6-dichlorophenol, 2,6-dichloroaniline and *O*-dichlorobenzene (P_2_) through reactive species attack and the C–N bond cleavage. In pathway b, 3,4-dichloropyridine can also be converted to *O*-dichlorobenzene during the oxidation process by losing NH_3_. In the pathway c, intermediates of 2,6-dichlorophenol, 2,6-dichloroaniline and *O*-dichlorobenzene can be formed by cleavage of C–N bond of the DCF by reactive species attack.

## Conclusion

In the present study, CoFe_3_O_4_-supported MWCNTs magnetic nanoparticles were synthesized using a co-precipitation method and were first used to activate the PMS and to degrade the DCF. The results of the comparative tests showed that the MWCNTs-CoFe_3_O_4_/PMS system has high catalytic activity in the degradation of DCF compared to other systems. This increase in efficiency was due to the capability of each material present in the MWCNTs-CoFe_3_O_4_ catalyst to activate PMS. The highest removal efficiencies of DCF (99.04%), COD (79.89%) and TOC (50.11%) were achieved at pH of 7, PMS dosage of 4 mM, catalyst dosage of 500 mg L^−1^, DCF concentration of 30 mg L^−1^ and time of 120 min. Meanwhile, anions (NO_3_^−^, Cl^−^ and HCO_3_^−^) showed the hindering effect on DCF removal and the order of hindering was HCO_3_^−^ > NO_3_^−^ > Cl^−^. The relationship between temperature and constant rate showed that the MWCNTs-CoFe_3_O_4_ catalyst requires less activation energy than other catalyst used in previous studies. In addition, the results of the scavenging experiment suggested that both 
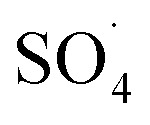
 and ·OH were produced in the MWCNTs-CoFe_3_O_4_/PMS system and 
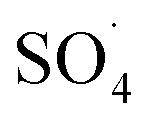
 was the dominant reactive species for DCF removal. The catalytic stability of MWCNTs-CoFe_3_O_4_ more than 4 times showed the reusability for long-term DCF catalytic degradation. The dichlorophenol, 2,6-dichloroaniline and *O*-dichlorobenzene were identified as the main intermediates by GC-MS analysis and the DCF degradation pathways were proposed based on the identification of intermediates. Finally, based on the high stability, low activation energy and easy synthesis, it can be concluded that MWCNTs-CoFe_3_O_4_ is a promising alternative for conventional heterogeneous catalysts for activating PMS in the catalytic degradation process of DCF.

## Conflicts of interest

There are no conflicts to declare.

## Supplementary Material
